# Reduced Physical Activity and Increased Weight Status in Children and Adolescents During the COVID-19 Pandemic: A Systematic Review

**DOI:** 10.3390/children12020178

**Published:** 2025-01-30

**Authors:** Luciana Zaccagni, Emanuela Gualdi-Russo

**Affiliations:** 1 Department of Neuroscience and Rehabilitation, Faculty of Medicine, Pharmacy and Prevention, University of Ferrara, 44121 Ferrara, Italy; emanuela.gualdi@unife.it; 2 Center for Exercise Science and Sports, University of Ferrara, 44123 Ferrara, Italy

**Keywords:** exercise, BMI, weight status, childhood, adolescence

## Abstract

Background/Objectives: The COVID-19 pandemic has impacted the lifestyles of children and adolescents because of the restrictions imposed to contain the infection. This systematic review examined the possible reduction in children’s and adolescents’ physical activity and changes in their BMI and weight status. Methods: A systematic review (PROSPERO: CRD42024589208) of English-language studies published up to 1 October 2024 in individuals with a mean age between 6 and 18 years that investigated physical activity data before and during or after the pandemic and focused on their BMI and weight status according to age, sex, and country making use of PubMed, Web of Science, and Scopus, identified 1040 possible articles. Results: Following the PRISMA statement, 26 articles with a whole set of 138,737 children and adolescents were enclosed in the review. The majority of included articles were of moderate quality, as determined by the Newcastle–Ottawa Scale adapted for observational studies. The studies found a decrease in PA, an increase in sedentary activities during/after the lockdown, and an increase in BMI and prevalence of overweight and obesity, especially in males and 8–11-year-olds. Conclusions: The interruption or restriction of outdoor physical activities and sports during the COVID-19 pandemic resulted in a more sedentary lifestyle among children and adolescents, contributing to an overall increase in BMI and obesity, with alarming implications for related health risks and other noncommunicable diseases. Therefore, the adverse effects of restrictive measures should be assessed when implementing public health strategies during pandemics.

## 1. Introduction

In the aftermath of the COVID-19 outbreak, most nations worldwide placed stringent restrictions on thwarting the spread of the infection. On 11 March 2020, the World Health Organization (WHO) proclaimed coronavirus 2019 (COVID-19) as an epidemic due to its fast diffusion worldwide.

Movement restrictions or lockdowns affected more than a third of the world’s population at the beginning of April 2020 [1]; China was the first nation to activate the lockdown in Wuhan as of 23 January 2020. Among the first European countries, Italy imposed a nationwide lockout starting 9 March 2020, where residents were ordered to stay at home; schools, sports facilities, restaurants, and stores (except grocery stores and pharmacies) were closed; there were travel restrictions; and isolation. Similar initiatives since followed elsewhere: over 100 nations implemented stringent measures to limit the transmission of the epidemic, awaiting the availability of vaccines and efficacious drugs [2].

As a result of the restrictions imposed during the epidemic [3], the daily routines of children and adolescents were disrupted, leading to changes in eating behaviors and sedentary lifestyles [4,5]. On the one hand, physical activity (PA) decreased due to the closure of schools, sports centers, and outdoor activities while sedentary and screen time increased [6]. Consequently, the WHO guidance for individuals aged 5–17 years to perform an average of 60 daily minutes spent in moderate to vigorous PA was disregarded during periods of isolation [7]. On the other hand, increased food intake including unhealthy food characterized this period, generally leading to increased body weight and health risks, as shown by studies conducted on adults, children, and adolescents [8,9,10].

Similarities between some of the referred changes (increased inactivity, school absence, etc.) and those that occur during the holiday season when there is a tendency for increased weight and adiposity can be pointed out [2,11,12]. However, there is no doubt that there were substantial differences between these periods from a psychological perspective: anxiety, depression, misbehavior, poor attention span, and impulsivity were observed in children, and especially adolescents during the epidemic, with a rising trend of mental health impact as the duration of isolation increased [13,14]. Conversely, summer vacations generally provide a chance for increased family interaction and beneficial recreational activities. However, this can also be a stressful time for low-income families, during which loneliness can hurt young people’s mental well-being [15]. Briefly, the extent of the restrictions applied during the epidemic and the rapidity of their application accentuated the critical situations already highlighted during the summer holidays, leading to a rapid weight gain that is difficult to shed [16,17].

Following the global health emergency brought about by the pandemic outbreak, there has been a collective effort to try to understand the biological, psychological, and sociological determinants of weight gain [18,19] because of the serious health risks caused by overweight/obesity. The obesity prevalence in childhood and adolescence has been increasing globally, reaching a plateau in most high-income countries around 2000 [20,21]. Recently, the COVID-19 pandemic has reportedly resulted in a weight gain in children and adolescents compared with the pre-pandemic rate as a result of lifestyle changes. Obesity during childhood tends to persist into adulthood. This condition is commonly combined with both psychosocial and cardio-metabolic comorbidities as well as early death [22,23].

Various determinants could have caused a gain of weight in children and adolescents over the course of the pandemic, and among them has emerged, in particular, the large reduction in PA. Sedentariness, measured as time spent on screen, is correlated with obesity risk factors in adolescents, and conversely, low sedentary and high PA levels are crucial for good health including weight status [24]. A recent review comparing the efficacy of several interventions on lifestyle for body composition in children showed that PA engagement is the most efficient tool in reducing the BMI, and the second most efficient in reducing the BMI z-score and body fat percentage [25]. In Europe, the decline in all kinds of PA recorded during the pandemic was greatest during periods when schools were closed [26]. The COVID-19 pandemic, with school closures, isolation, and the subsequent physical inactivity of children and adolescents, represents an unprecedented health crisis whose extent has yet to be fully understood.

Although a general increase in weight and obesity during the pandemic was detected in children and adolescents, further research is needed to fully understand the effect of lifestyle changes. Notably, the contribution of this review differs from that of several previous studies [27,28,29] in that it intends to systematically explore the literature focusing on a precise factor (PA) instead of generic unhealthy behaviors or eating habits [6,8,29]. Although the epidemic and subsequent restrictions affected almost all populations, some reviews only focused on specific geographic areas [26,30]. In addition, we selected the articles to be included regardless of the type of study design, unlike others (e.g., Anderson et al. [10] who only included longitudinal studies) focusing on changes in BMI and weight status, while most studies mainly considered weight gain [2,16,17], although only the ratio of weight to height (BMI) allows individuals to be categorized into different weight categories. The application of this anthropometric index is important, especially in growing subjects, because of the significant changes in stature with age. Finally, early reviews published on the topic [2,27,28], although essential in highlighting the early trends observed at the beginning of the pandemic, do not allow for an assessment of the possible evolution of the phenomenon throughout the pandemic period.

Taking the above into account as well as some inconsistent findings from the literature related to the COVID-19 outbreak [2,29,31,32], the main purpose of this review was to respond and provide up-to-date evidence to the following questions: What is known about the PA decline during the lockdown in response to the outbreak of COVID-19? What is known about changes in the BMI and weight status of children and adolescents? What is known about the possible trends of these traits with age, sex, and geographic area? Understanding these features is critical for targeting future interventions under similar isolation conditions in growing individuals.

## 2. Materials and Methods

Our systematic review was conducted following the PRISMA recommendations [33]. In the Supplementary Materials, we include Table S1 with the PRISMA 2020 checklist for abstracts and Table S2 with the PRISMA 2020 checklist. The review was registered in PROSPERO with the protocol CRD42024589208.

### 2.1. Search Strategy and Selection Process

We conducted a systematic search for articles in the PubMed, Web of Science, and Scopus electronic databases from 1 January 2020 to 1 October 2024 using the following string: (quarantine OR confinement OR lockdown OR lock-down OR isolation OR social-distancing) AND (“physical activity” OR “physical inactivity” OR exercise OR sedentar*) AND (covid* OR sars-cov-2 OR coronavirus OR pandemic) AND (child* OR schoolchildren OR adolescen* OR boys OR girls OR students) AND (“body mass index” OR BMI OR “weight status” OR underweight OR “normal weight” OR overweight OR obes*). We restricted the search outputs using publication date filters for the three databases and the age filters “child: 6–12 years” and “adolescent: 13–18 years” on PubMed.

A manual and independent review of potential articles for eligibility was performed by both authors (L.Z.; E.G.-R.) in two steps, on the basis of: (1) the title and abstract; (2) the full text. Specifically, after eliminating duplicates, the two authors, in line with the PRISMA guidelines, proceeded to independently examine the consistency of the articles with the inclusion and exclusion criteria reported below: the titles and abstracts of articles were screened for relevance and eligibility. The full text of the articles that passed this screening was examined, recording any reasons for exclusion. Any doubts or disagreements about the eligibility of the selected articles were cleared up by additional examination and discussion between the co-authors. At the end of the selection process, the bibliographies of the included articles were examined to check for any other articles not in the electronic databases considered.

### 2.2. Inclusion and Exclusion Criteria

Eligibility criteria were selected according to the PECO framework [34] considering the population, exposure, comparator, and outcomes. In this research, the population consisted of individuals aged 6 to 18 who suffered from being socially isolated during the COVID-19 outbreak with the resulting restrictions on sports and PA with no geographical limitation. The COVID-19 pandemic constituted the exposure. Comparisons were, firstly, trends between the pre-COVID and during/post-lockdown, and secondly, trends with age, sex, and geographical area. Primary outcomes concerned the effects of PA practice on body characteristics (BMI and/or weight status) over the periods considered. Secondary outcomes concerned the influences of age, sex, and population factors. In addition, only peer-reviewed, English-language articles, whose full text was available, were included.

The following exclusion criteria were applied: (i) mean sample age below 6 years or above 18 years; and (ii) lack of pre- and during/post-lockdown comparison data for PA (or sedentary activities) and BMI or weight status. We excluded studies that included the same sample or a sub-sample that had already been reported in another article. We also excluded qualitative studies, studies of clinical populations (including obese patients), non-English articles, literature reviews, editorials and commentaries, letters, abstracts, reports, case studies, books (or book chapters), protocol studies, and conference proceedings. The entire selection process and exclusion reasons are specified in the Results section using a PRISMA flowchart.

From each selected study, the two authors independently extracted the following information if available: the name of the first author and year of publication, study design, sampling period and country, study sample (size, sex, age), sample characteristics (changes in PA, BMI and/or weight status), and the main outcomes. We reported a qualitative summary of the data in the tables and listed the studies in alphabetical order.

### 2.3. Quality and Risk of Bias Assessment

Consistent with the PRISMA guidelines for systematic reviews [33], the two authors conducted an independent evaluation of all included investigations with the adapted Newcastle–Ottawa Scale (NOS) for observational studies [35,36].

This tool includes four main assessment criteria (study clearness; sample selection; comparability; outcome). Concerning the sample selection criteria, we assessed “Exposure assessment” regarding PA, while for “Assessment of the outcome”, we focused on the tools used in the body weight evaluation. Further adaptations of the NOS involved the “Clearness of the aim”, in which we decided to assign a score of 2 to the accuracy and relevance of the question in light of the available literature when the number of citations was ≥10, and 1 when it was <10. We also maintained a score equal to 0 on the NOS-adapted scale for the third item in sample selection relative to the non-respondents category when “One or none of three” of the possible conditions were fulfilled, as already undertaken in previous reviews [37,38]. Finally, regarding the “Comparability of individuals from different groups based on study design or analysis”, we assigned a score of 1 when the sex composition of the sample was the same, or in any case, did not differ more than 10%.

We calculated each study’s overall score (range: 0–16) by summing the ratings of each component. We rated studies with scores of 13–16 points as high quality and low bias risk (scores > 75%), studies with 9–12 point scores as of moderate quality and risk (scores > 50%), and studies with ≤8 point score (scores ≤ 50%) low quality and high risk [36]. To ensure transparency, no study was dropped from this review due to the results of this assessment. Again, any discordant ratings between the two authors were addressed through discussion.

## 3. Results

### 3.1. Study Selection

**Figure 1:**
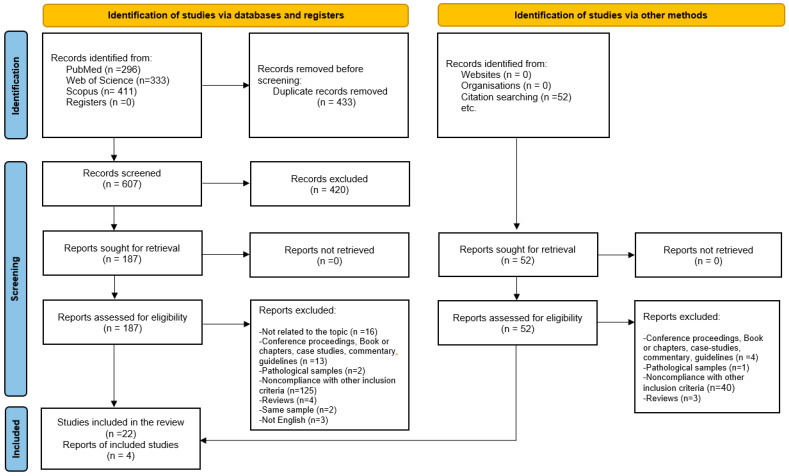
Flowchart of the study selection process (PRISMA 2020 flow diagram).

The search across the three databases (Web of Science, Scopus, and PubMed) identified 1040 records. After removing 433 duplicates, we screened the titles and abstracts of the remaining 607 articles and excluded 420 non-relevant articles. We then screened the full text of the 187 articles and excluded 165, identifying 22 articles that met the inclusion criteria. The screening of the quotations from the 22 selected articles led to the inclusion of four additional articles, so in this systematic review we considered 26 articles. Figure 1 displays the flow diagram of the whole selection process of the study.

### 3.2. Study Characteristics

Table 1 and Table 2 synthesize the characteristics of the enclosed papers about PA change and BMI/weight status change caused by lockdown in children and adolescents.

Half of the articles were published in 2022, seven in 2021, four in 2024, and one each in 2020 and 2023. These studies were performed in nineteen different nations: thirteen studies were performed in Europe (including four in Italy, two in Germany, and one each in England, Croatia, Spain, the Netherlands, Ukraine, the Czech Republic, and Poland), Eleven studies in Asia (including four in China and one each in Jordan, Saudi Arabia, Qatar, Iran, South Korea, Singapore, and India), one in South America (Argentina), and one in North Africa (Algeria).

Seven studies (27%) used a longitudinal design [43,45,47,51,54,60,63], two studies (8%) used both a longitudinal and a cross-sectional design [42,59], and the majority (the remaining seventeen studies equal to 65%) used a cross-sectional design. When the sample was observed more than once, the first time was before the COVID-19 emergence, usually in the period October 2019–March 2020, [42,43,45,47,51,54], and the second time was during the pandemic one year later [42,43,45,51] or before, in June–July 2020 [47,54]. Azrak et al. [42] and Palmieri et al. [51] carried out a third data collection another year later in September–November 2021.

The participants in these studies were healthy children and/or adolescents of both sexes, mostly primary, secondary, or high school students. The smallest sample size was 50 Spanish students from a primary school in Cantabria (Spain) [54], and the largest was 112,251 Korean students from 800 secondary and high schools [52]. Specifically, regarding the number of subjects studied, four studies (15%) had a sample size of less than 100, twelve studies (46%) had a sample size between 101 and 1000, nine studies (34.6%) had a sample size between 1001 and 10,000, and only one study (4%) had more than 10,000 subjects. Regarding the age of the subjects, six studies (23.1%) considered children under the age of 10, twelve studies (46.2%) included the age range 10–15, two studies included adolescents over the age of 15, and six studies (23.1%) included subjects in the full age range of 6–18.

### 3.3. Analysis of Physical Activity

PA was measured objectively in only a few studies, using an activity monitor (accelerometer Actigraph wGT3X-BT delivered to the doorstep or delivered to the school) [56,59] or through personal devices (smartwatch or smartphones) reporting the daily number of steps [57]. In most studies, the amount of PA of the children and adolescents during the lockdown was assessed subjectively, self-reported, or reported by parents using validated questionnaires (GPAQ [64], PAQ-C [55,57], Baecke [59], or IPAQ long form [62]) or by answering simple questions (like “Did you do any PA before lockdown? and “Did you do any PA during lockdown?”) [51], the mean time spent in sports clubs [43], the proportion of subjects that never engaged in PA during weekdays [41], the proportion of subjects practicing PA on a determined number of days per week [39], MVPA ≥ 60 min per day on all 7 days, and the number of days in the last week with MVPA ≥ 60 min [50]). PA was assessed both objectively using an Actigraph accelerometer and subjectively using the PAQ-C questionnaire in two studies [45,60]. Regardless of the method used to assess PA, all studies found a decrease in PA in children and adolescents during or after the lockdown, and when considered, an increase in sedentary time and a lower proportion of subjects meeting the WHO recommended minimum of 60 min per day of moderate-to-vigorous physical activity (MVPA) in the students under lockdown or studying at a distance [64].

Specifically, Abed Alah et al. [39] found a significant 20% decrease in the proportion of Qatari children and adolescents who engaged in regular sports before and during COVID-19, and that 35% fewer students engaged in PA 2–4 days per week and 37% more engaged in PA once or less per week. Al-Agha et al. [41] found a 20% increase in males and a 16% increase in females in the percentage of Saudi subjects who never engaged in PA activities during COVID-19 compared with before. In He et al. [47], Chinese children decreased their daily PA by 7.3 min (equivalent to 8%) and increased their screen time by 15 min (equivalent to 17.6%). In Europe, Croatian students [48] with low PA levels increased by 57%; Spanish students who did not engage in any PA increased by 28% [54]; 75% of German students reduced their participation in sports and 55.8% increased their sedentary behavior [55]; 44% of another German sample [61] reduced their PA (especially the min 10–12 age group by 57%); 62% of Dutch students reduced their PA during lockdown; almost 15% of English children stopped participating in a sports club [43]; in Italian students [45], the MVPA decreased by 19.5 min per day in boys and 8.3 min per day in girls: as a result, the prevalence of boys not meeting the recommended levels of PA increased by 41% and that of the girls by 14%.

### 3.4. Analysis of BMI/Weight Status

The methods used to assess the BMI were the direct measurement of weight and stature by expert personnel [43,47,48,51,54,55,58,59,60,63] or the retrieval of data from electronic health record systems (especially for the pre-COVID-19 data) [39,41,42] or from school records [49] or online questionnaires with self-reported data on weight and stature from which the BMI was calculated [40,44,52,53,57,62,64]. The BMI values were either expressed in raw kg/m^2^ or transformed into BMI-for-age Z-score and then used to establish the weight status by classifying subjects as underweight, normal weight, overweight, or obese on the basis of age- and sex-specific BMI thresholds. In some studies, weight status was asked and then self-reported by the parents or the subjects themselves [46,61]. Regardless of the method of BMI/weight status assessment, all studies observed increased BMI values and the prevalence of overweight/obese subjects. In particular, the zBMI change ranged from 0.07 in Chinese children [56] to 0.9 in Iranian adolescents [49], while the BMI change ranged from 0.1 kg/m^2^ in the Chinese sample [47] to 1.5 kg/m^2^ in English children [43] and Singaporean children [58]. When the change was considered according to sex and age, it was usually greater in males and the 8–11 year age group or in the youngest age group considered [39,47,49,52]. Unlike the general trend, Łuszczki et al. [50] found a lower (−0.32 kg/m^2^), although not significant, BMI (with a halving of the percentage of obese subjects from 11.5% to 6.4% and an increase in underweight subjects (+3.3%)), despite lower PA during the pandemic than before: anthropometric data before the pandemic were directly measured but during the pandemic were self-declared by the parents or themselves. Similarly, Štveráková et al. [57] found a decrease in BMI (−0.57 kg/m^2^) despite reduced PA (−0.38 in PAQ-C score): even in this study, the anthropometric data were directly measured before COVID and self-reported during the COVID pandemic.

The perception of the weight status of their child by Italian parents in the study by Farello et al. [46] was also in agreement with what has already been established about directly collected data: they observed a higher number of reports post-lockdown of overweight (+5.7% in the 5–11 age group and +0.8% in the 12–18 age group) and obesity (+0.6% in the 5–11 age group and +0.7% in the 12–18 age group) as well as a lower number of reports of normal weight or underweight after the closure.

To look for possible predictors of changes in the BMI or BAZ scores over the period of COVID-19-related school closures, the authors of some of the papers included in this review performed different statistical analyses (univariate linear regression, multivariate linear regression, or logistic regression) using different independent and dependent variables. The linear regression performed by Abed Alah et al. [39] showed that the PA reduction while schools were closed was a significant predictor of the increase in BAZ scores in different sexes and age groups. In the study of Kenđel Jovanović et al. [48], the odds of overweight and obesity before lockdown were significantly lower for the ones with moderate PA than for the ones with low PA (OR = 0.71; *p* = 0.016). Those who played organized sports between two and three times per week were less likely to be overweight and obese than those who committed to less than two times a week (OR = 0.59; *p* = 0.001). During the lockdown, the odds for overweight and obesity increased almost twofold (OR = 0.23, OR = 0.36; both *p* < 0.001) for those engaged in organized sports more than two days a week than those who played sports less than two days a week. Generalized estimating equation analysis performed by Yang et al. [63] reported that the teen students involved in MVPA for at least 60 min per day for all seven days were less likely to have obesity. In addition, boys with ≥2 h of computer time per day and girls with ≥2 h of mobile screen time per day or ≥2 h of TV time per day were more likely to have obesity. Similar results were found by He et al. [47] in their generalized model showing that less daily PA and more screen time were positively related to obesity.

### 3.5. Risk of Bias

The range of NOS scores of the included articles was from 6 to 14 points: the lowest score was 6 in the longitudinal study on Spanish students [54], and the highest score of 14 was obtained by two longitudinal studies, one on Indian students [60] and one on Chinese students in Shanghai [63]. Most studies (65.4%) were of moderate quality, 23.1% were of low quality, and 11.5% were of high quality. The highest scores were obtained in the items: clarity of the stated aim, assessment of the outcome, and statistical tests (no study obtained a score of 0); the lowest scores were obtained in the items “non-respondents” (20 studies obtained a score of 0) and “control of confounding factors” (Table 3).

## 4. Discussion

In this review, we examined the impact of the restrictions imposed during the COVID-19 pandemic, such as the lockdown, which disrupted daily routines by suspending school attendance and physical activities including outdoor activities and sports, increased screen time, and home confinement [5,65]. The same sleep–wake rhythm and eating habits, with large use of comfort food, also changed during this period [6,66]. As a result of the restrictive measures implemented by most countries around the world, on 7 April 2020, 3.9 billion people, over one-half of the population of the world, were in lockdown [67].

Our results suggest that the COVID-19 pandemic, characterized by drastic measures of social estrangement and confinement, significantly affected the practice of PA and the weight status and BMI of children and adolescents.

In particular, through this systematic review, we reported data regarding a total of 138,737 individuals with an average age between 6 and 18 years, distributed across 19 countries from 4 continents worldwide (Algeria, Argentina, China, Croatia, Czech Republic, England, Germany, India, Iran, Italy, Jordan, Qatar, Poland, Saudi Arabia, Singapore, South Korea, Spain, the Netherlands, Ukraine).

The practice of PA is essential for children and adolescents to develop social relationships and reduce the risk of psychological, social, and physical issues [18,68]. In this period of growth, characterized by physical and mental fragility, the abrupt interruption of social contacts and life habits imposed during the epidemic period by the lockdown, with the suspension of PA among other things, seems to have affected physical and mental health. According to this review, the highest percentage decrease in PA was observed in children from Europe (Italy and Spain), the highest overweight/obesity prevalence in Qatar and Argentina, and the highest increase in BMI according to z-BMI in the Middle East (Jordan, Qatar, Iran), in addition to England and Singapore (BMI values). Notably, some Eastern European countries (Ukraine and Poland) showed no change or increase in BMI after the pandemic. These trends may depend on the sample composition examined (changes were greater in children/pre-adolescents than in adolescents and males than in females) and on the spread and severity of the infection with consequent differences in the restrictions imposed by governments. Thus, China was the first to impose a strict lockdown on 60 million people in Hubei Province starting in January 2020, followed in March 2020 by Italy with 67 days of lockdown [69]; the longest lockdown period in 2020 with 234 days was implemented in Argentina [70], unlike some countries where this strategy was never implemented (e.g., Sweden among European countries). Specifically, in Sweden, where mild restrictions were implemented, Helgadóttir et al. found, in an accelerometer-based study on a large sample of Swedish adolescents, that the MVPA remained generally stable (even increased significantly by 4 min during weekdays) while the light PA and sedentary time changed unfavorably, especially in girls and overweight/obese subjects [71].

### 4.1. Physical Activity During the Lockdown

Although it is generally believed that PA is practiced inadequately compared with general indications [7], a further negative trend in PA practice was observed from childhood to adolescence during the epidemic. Therefore, this decrease in PA is worrisome for child and adolescent health: the reduction in the practice of PA, already deemed inadequate according to the WHO recommendations for 5- to 17-year-old children/adolescents, can lead to repercussions on the short-term, medium-term, and long-term physical and cognitive development, with a tendency to weight gain, cardio-metabolic, and psychological problems [72]. This review showed that the decrease in PA during lockdown was larger in children/pre-adolescents and the male sex. Adolescents are generally more prone to sedentariness than children because of different age-typical interests, which in the lockdown period manifested in more screen time devoted to the Internet and gaming [73]. As for sex, it is well-known that girls usually engage for fewer hours than boys in PA during the week and on weekends [72,74,75]. Therefore, the further decrease due to the pandemic in the amount of time devoted to PA practice by girls is worrying. The female sex is less physically active than the male sex throughout their life span, through childhood, adolescence, and adulthood [76]. Girls face greater barriers than boys in participating in sports: their cultural background, dissatisfaction with their physical appearance, and athletic ability are some of the possible causes [77]. During the lockdown, screen time increased, contravening the WHO recommendation of a maximum of 2 h per day for children and adolescents [7,72]. Intensive Internet use on cell phones and video games is linked to increased BMI values and overweight in adolescents [78], and Internet addiction is more pronounced in females than in males [73]. The concept that practicing PA increases health benefits and decreases the risk of problematic Internet use [79] is particularly valid in the case of confinement as during the recent pandemic [80]. In general, Internet use is positively linked to an increased probability of overweight and obesity [81].

### 4.2. BMI/Weight Status During the Lockdown

The increase in the BMI mean values and overweight/obese prevalence was mainly achieved in children/pre-adolescents and the male sex, as reported above. The increase in BMI and weight status is generally justified in the literature by the restrictions imposed during the outbreak of COVID-19 (among others: [10,82,83]). Schools were shut down during the COVID-19 pandemic, and most scholastic and extracurricular physical activities and sports were suspended. While a regular practice of PA, in an indoor or outdoor space sufficient, may help to reduce depression and anxiety development due to epidemic stress [18,84]), it would have been crucial in controlling weight gain caused by a sedentary lifestyle and jungle food use during the confinement period, especially at the ages considered. According to a previous review [66], the greater influence of lockdown on weight gain in pediatric age compared with adulthood must be highlighted. Regarding weight status, a particular rise in overweight prevalence was observed, while obesity tended to remain stable. This trend is probably related to the greater sedentary behavior of the obese even before the lockdown, which therefore did not particularly affect them from this point of view. The weight gains observed during the epidemic period are believed to be similar to those resulting from physical inactivity during summer vacation [2,12]. However, some apparent differences should be pointed out: the stay-at-home period during the epidemic was coercively imposed on the entire population and has resulted in mental health effects (anxiety, depression) related to both losses of individual freedom of movement and socialization, and concern about getting sick that affected the behavior from sedentary to emotional eating (snacks and sweets are considered the most common forms of psychological compensation) [85]. Future studies may help verify whether the bad habits acquired during the confinement period, where, unlike holiday-related effects, the lockdown period could have long-lasting physical and mental health effects: recent European studies seem to indicate maintenance in the post-COVID period of negative effects on the BMI and weight status despite the resumption of the practice of PA [55,83,86].

### 4.3. Strengths and Limitations

The main strengths of this review are as follows: adherence to the PRISMA statement; an accurate literature search through a specific string aimed at identifying as many relevant articles as possible, complemented by the final search on the bibliographies of the selected articles; an independent evaluation of the eligibility, selection process, and data extraction for the study by the two authors; and a quality evaluation of all studies involved. Assessment of the appropriateness of the inclusion/exclusion of studies in the review was also carried out through direct contact with the authors to avoid overlapping sampling.

This review had some weaknesses. We first limited ourselves to examining the increase in BMI and weight status during lockdown, assuming that it depended on decreased PA. However, numerous factors may have played a role in this trend, but the analysis of other factors was beyond the scope of the study. Furthermore, our review showed that studies on PA and BMI conducted in different populations obtained concordant results.

The studies were performed at different epidemic and pre-epidemic times. The extent of restrictions applied in various nations worldwide, such as the length of the lockdown, was not necessarily the same, depending on the policies adopted and the severity of the epidemic. In addition, a further limitation came from the studies’ heterogeneity not only in the size of the sample, gender, and ethnic group but also in the variables analyzed (from the frequency in PA practice to the measurement of precise parameters with the accelerometer) and methods (direct measurements vs. interviews; direct participant analysis vs. parental interview). Enhancing the heterogeneity, most studies used a cross-sectional design, but nine included studies were conducted with longitudinal or mixed designs, resulting in possible different levels of reliability of the results obtained. However, the trend of the examined characters was the same regardless of the study design employed. In addition, other methodological differences consisted of using reliable medical databases in retrospective studies, while others employed self-reported data, resulting in an increased risk of bias. Even BMI, sometimes based on direct measures of weight and height, and others on participant- or parent-reported data, is referred to as kg/m^2^ values or z-scores. Moreover, the resulting weight status was categorized according to thresholds in the WHO growth curve for children or Cole cut-off values. Concerning the percentiles, the reference for underweight was, according to different studies, a BMI ≤ 2, ≤5, or ≤10 percentile, for overweight ≥85 or ≥90 percentile, and for obesity ≥95 or ≥97 percentile. Comparisons between the resulting frequencies must therefore be made with extreme caution. Finally, the statistical parameters related to the association between PA and BMI, even when present among the same variables, differed, ranging from the Pearson’s r coefficient to odds ratios up to multiple linear regression between an independent variable (generally BMI or z-BMI) and two or more dependent variables. The generality of the selected articles did not consider potential confounders in their analyses. Further limitations of this study can stem from the limitations of the studies reviewed. Precisely because of the heterogeneity of the examined studies, we could not undertake a meta-analysis and merely reported the results with a narrative synthesis. Finally, only articles in English were included in our review excluding articles published in other languages.

## 5. Conclusions

Our study showed that a reduction in PA and an increasing BMI and weight status consistently characterized children and adolescents undergoing lockdown in different countries. Although a growing trend of overweight/obesity is evident in the world, the COVID-19 epidemic seems to have worsened the situation with particular reference to children. In the case of an epidemic, policymakers, educators, and health professionals should carefully evaluate the negative impact of restrictions and social isolation on children and adolescents, developing effective strategies to promote healthy development and growth.

## Figures and Tables

**Table 1:** Characteristics of the included studies (in alphabetical order).

Author (Year) -Study Design-	Country (setting)	Period and Method of Data Collection	Sample Size	Age (years) Range; M ± SD	Method of PA Assessment	Method of Stature and Weight Collection for BMI (WS assessment)
Abed Alah et al. (2024) [39] -cross-sectional-	Qatar	June–August 2022 Telephone interviews with parents.	N = 1546 (F = 50.3%) Children (8–11 yrs): 54.7% Adolescents (12–15 yrs): 45.3%	Range: 8–15 Mean age: 11 ± 2	Questionnaire: Number of days in a typical week (from 0 to 7 days) before and during COVID the child did a total of 60 min or more of PA, which was enough to raise his/her breathing rate	Retrieved from the electronic health records system (values > +1 SD represent OW (or BMI for age more than 1 SD above the median) and >+2 SD represent Ob (or BMI for age more than 2 SD above the median)).
Al Hourani et al. (2022) [40] -cross-sectional-	Jordan	15–30 June 2020 Questionnaire (Google Form) to parents of children or directly to adolescents.	N = 447 (F = 51.6%) Children (6–12 yrs): 51.4% Adolescents (13–17 yrs): 48.6%	Range: 6–17	Questionnaire: Amount of PA (h/day)	Self-reported (OW and Ob: values > +1 SD represent OW (equivalent to BMI 25 kg/m^2^ at 19 y) and >+2 SD represent Ob (equivalent to BMI 30 kg/m^2^ at 19 y). WHO reference curves, 2007)
Al-Agha et al. (2022) [41] -cross-sectional-	Saudi Arabia Pediatric Endocrine Clinic at King Abdulaziz Hospital, Jeddah.	Phase 1: from September 2019 to January 2020 (pre-COVID-19) Phase 2: From January 2020 to May 2020. Phase 3: from September 2020 to May 2021. Direct interviews with participants and parents	N = 518 (F = 50.4%) Primary school (54.4%), intermediate school (33.2%), secondary school (12.4%)	Range: 6–18	Questionnaire: Amount of daily time spent exercising	From hospital records (NW: 5th–85th p. OW: 85th–95th p, Ob > 95th p, severely Ob > 99th p using age-stratified Center for Disease Control and Prevention growth charts)
Azrak et al. (2022) [42] -cross-sectional and longitudinal-	Argentina Healthy Children Outpatient Service (La Plata)	Pre-COVID Historical Control Group (HCG): 1 December 2019–10 March 2020. Post-lockdown Group (LG): 1st visit on November 2020 and 2nd visit on September 2021 in addition to pre-pandemic measurements (baseline) Questionnaires filled out by parents	LG: N = 144 (F = 50%) HCG: N = 134 (F = 57.7%)	6–9 yrs at the beginning of the pandemic Age means at baseline: LG: 6.74 HCG: 6.73	Questionnaire: Changes in PA (less, the same, more)	From hospital records for HCG and directly measured at the first and second visits for LG (Based on BMI values converted in z-scores using WHO Anthro-Plus software version 1.0.4. A zBMI > 1 was defined as OW, and a zBMI > 2 as Ob, according to the WHO)
Basterfield et al. (2022) [43] -longitudinal-	England Primary school of a deprived area in North-East England	October 2019 (baseline) and 10 November–1 December 2020 (follow-ups). Questionnaires were self-completed by participants.	N = 178 (F = 46.6%) 94% of the sample was from the White ethnic majority	Range: 8–10 Mean age at baseline: 9.1 ± 0.6	Questionnaire: Total time spent in sports clubs per week was reported through Leisure Time Physical Activity Survey	Directly measured (BMIz relative to Cole cut off values were calculated using LMS Growth Excel add-in and presented as a z-score (i.e., standard deviations from the age- and sex-normative mean) to assess weight status: UW: ≤2nd centile; healthy weight: >2nd <85th centile;OW: ≥85th <95th centile; Ob: ≥95th <99.6th centile; severely Ob: ≥99.6th centile)
Benmerzoug et al. (2022) [44] -cross-sectional-	Algeria	April–May 2020 and 11 July–10 August 2020 Online questionnaires filled out by parents	N = 275 (F = 48.4%)	Range: 5–12 Mean age: 8.62 ± 2.28	Questionnaire: Frequency and duration of PA as sports practice, regular activity, and playing time before and during L	Self-reported
Dallolio et al. (2022) [45] -longitudinal-	Italy Primary school in a northern Italian city (Imola, Emilia Romagna Region).	1st survey: October 2019 2nd survey: January 2021 (after 1 year of pandemic) Online questionnaire	N = 77 (F = 37.7%) This is a randomized sample from the I-MOVE study	1st survey: 7.84 ± 1.41 2nd survey: 9.19 ± 1.41	PA was assessed in both surveys by self-reported (PAQ-C) and objective measures (accelerometer) Light, moderate, and vigorous PA per day was calculated using cut points by Evenson	Directly during 1st survey, reported by parents during 2nd survey (WS according to Cole cut off values by sex and age)
Farello et al. (2022) [46] -cross-sectional-	Italy	Data collection: 7–18 January 2021 Period investigated: the first Italian L (9 March–18 May 2020) Online questionnaire filled out by parents	N = 965 (F = 45%) Children (5–11 yrs) N = 402 (52.4%) Adolescents (12–18 yrs): N = 563 (39.7%)	Mean age: 12.28 ± 3.75 children: 8.45 ± 2.02 adolescents: 15.02 ± 1.80	Questionnaire	Perceived WS reported by parents
He et al. (2022) [47] -longitudinal-	China Primary and secondary schools in Chengdu	23 December 2019–13 January 2020 (before the pandemic) 16 June 2020–8 July 2020 (during the pandemic, when schools reopened in China) Self-administered questionnaires	N = 5963 (F = 50.1%) 80.6% of sample from primary school	10.7 ± 2.2	Questionnaire: Amount (min/day) of PA before and during pandemic	Directly measured by professional technicians from community hospitals. (WS was assessed based on age- and sex-specific BMI thresholds in the WHO growth curve for children)
Kenđel Jovanović et al. (2021) [48] -cross-sectional-	Croatia Schools in Primorsko-Goranska County	School year 2020/2021 Self-administered anonymous electronic survey.	N = 1370 (F = 53.4%) 11 yrs group: 18.0%, 12–13 yrs group: 51.9% 14–15 years group 30.1%	Range:10–15 Mean age M + F: 12.72 ± 1.17 M: 12.83 ± 1.18 F: 12.61 ± 1.15	Questionnaire: Total weekly PA (MET-min/week) before and during L Category of PA: (vigorous PA = 8.0 METs, moderate PA = 4.0 METs, walking = 3.3 METs, sitting = 2.5 METs, sleeping = 0.95 METs). PA level: -low (<600 MET-min/week) -moderate (600–3000 MET-min/week) -high (>3000 MET-min/week).	Directly by physical education teachers in September 2020 (the beginning of the school year) and May 2021 (WS was defined by centile grids for BMI according to age, gender, and height from “Croatian Anthropometric Reference Values for School Children and Youth”)
Khamesan et al. (2024) [49] -cross-sectional-	Iran Primary and junior middle schools in Bandar Abbas City	April–May 2022 Questionnaire.	N = 100 (F = 64%) -0–12 yrs group: 11% -12–14 yrs group: 57% -14–16 yrs group: 32%	Range: 10–16	Questionnaire: Frequency of 4 categories of PA before and during L: -sedentary activity -light intensity -moderate intensity -vigorous intensity	Collected from school records, as they were measured directly before and after the school closure at two time points, January–February 2020 and April–May 2022 (WS was based on z-BMI, which was calculated using a WHO growth chart taking into account children’s age and sex)
Łuszczki et al. (2021) [50] -cross-sectional-	South-East Poland Randomly selected schools in the Podkarpackie, Voivodeship	Pre: February–March 2020 During: 1 February–2 March 2021 (online questionnaire: 6–12 yrs filled by parents; 13–15 yrs filled on their own)	Pre: N = 376 (F = 50.3%) During: N = 640 (F = 51.9%) Total: N = 1016 (F = 51.3%)	Range (6–15) Pre: 10.51 ± 2.13 During: 10.79 ± 2.02	Questionnaire: Number of days in last week with PA ≥ 60 min before and during L	Pre: directly During: self-reported (WS defined in Polish BMI centiles) .
Palermi et al. (2022) [51] -longitudinal-	Italy Employers’ children in the Ferrari company based in Maranello (Mo), in the Emilia Romagna region	Annual screening visits in November 2019, 2020, and 2021. The questionnaire was filled out by parents in November 2020, after the first wave of the pandemic with the most rigorous lockdown rules in Italy.	N = 307 (F = 46.9%)	Range: 8–15 Mean age in 2019: 10.1 ± 2.3	Questionnaire: PA defined by 2 questions: Did you do PA before lockdown? Did you do PA during lockdown? Answer: Yes/No	Directly (BMI percentiles were calculated using the AnthroPlus software released by WHO)
Park and Lim (2022) [52] -retrospective cross-sectional study-	South Korea Use of the Korea Youth Risk Behavior Web-based Survey 15th and 16th raw data from 400 middle and 400 high schools	15th cycle: 13 June 2019–12 July 2019. 16th cycle: 3 August 2020–13 November 2020. Web-based, self-administered questionnaire.	15th cycle: N = 57,303 representing 2,673,152 students 16th cycle: N = 54,948, representing 2,683,547 students	Range: 12–18	Questionnaire: Frequency of subjects with at least 3 times/week of moderate PA > 60 min/day or vigorous PA > 20 min/day	Self-reported (WS was classified as percentile according to sex- and age-specific BMI on the 2017 Korea National Growth, classifying as Ob a BMI ≥ 95th percentile, OW a BMI ≥ 85th and <95th percentile, and NW a BMI < 85th percentile according to Chart for Children and Adolescents)
Pujia et al. (2021) [53] -cross-sectional-	Italy two cities: Brescia (North Italy) and Catanzaro (South Italy)	10 September 2020–19 April 2021 Online questionnaire filled out by parents	N = 439 (F = 44%) 5–9 yrs: 58.1% 10–14 yrs: 41.9%.	Range: 5–14 Mean age: 8.8 ± 3.0	Questionnaire: PA changes during the quarantine: more sedentary lifestyle, less sedentary lifestyle, did not know	Self-reported (Used the definition of obesity proposed by the Childhood Obesity Working Group of the International Obesity Task Force)
Ramos-Álvarez et al. (2021) [54] -longitudinal-	Spain Primary school in Cantabria	Pre-L: -1st: 14–18 October 2019 -2nd: 2–6 March 2020 Post-L: -3rd: 8–10 June 2020 Parents filled out a questionnaire.	N = 50 (F = 34%) Urban setting: 56%	Range: 11–12 Mean age: 11.40 ± 0.50	Questionnaire: PA as times/week	Directly
Samigullin et al. (2024) [55] -cross-sectional-	Germany Schools in the Rhein-Neckar region	October 2021–July 2022 (there was an interruption due to lockdown and high COVID-19 incidences). Parents filled out a questionnaire	N = 256 (F = 52%) 135 before and 125 after the interruption	Range: 7.1–9.3 Median age: 8.0	PAQ-C questionnaire, filled out by child with a study group member; questionnaire completed by parents, number of days with over 60 min of PA	Directly (OW is defined as BMI above the 90th percentile; Ob as BMI above the 97th percentile and underweight as below the 10th percentile).
So et al. (2022) [56] -cross-sectional-	Hong Kong (China) Schools in 5 districts	3-time points: -pre-pandemic (September 2019–January 2020) -during school closures (March 2020–April 2020) -Schools Partially Reopened (October 2020–July 2021) Questionnaire completed by a parent	Independent samples in each period Pre: N = 577 (F = 58%) During: N = 146 (F = 68%) Schools Partially Reopened: N = 293 (F = 77%)	Pre: 12.85 ± 2.61 During: 12.14 ± 2.90 Schools Partially Reopened: 11.93 ± 2.11	PA measured objectively with a wrist-placed activity monitor Chandler Cut-offs for PA and sedentary behavior were used to quantify the time spent as sedentary (VM < 3672 per min), engaged in light PA (3672 ≤ VM per min < 9816), moderate PA (9816 ≤ VM per min < 23,628), and moderate-to-vigorous PA (MVPA) (VM per min ≤ 23,628)	NR
Štveráková et al. (2021) [57] -cross-sectional-	Czech Republic	Data collected during L from November 2020 to January 2021 Data pre-Covid: collected in 2019 and previously published norms Completed the questionnaire either electronically or as a hard copy	N = 98 (F = 57.1%) Data Pre-Covid N = 206 (F = 48.5%)	Range: 8–12 M: 10.21 ± 1.49 F: 10.02 ± 1.46 M + F: 10.10 ± 1.47 Data Pre-Covid M: 11.08 ± 0.84 F: 11.17 ± 0.82 M + F: 11.13 ± 0.83	PA Questionnaire for Older Czech Children (PAQ-C/cz) During L, 35 children also reported a daily number of steps measured by smart watches or smartphones. (print screen from the device to prove the number of steps for each day)	Self-reported
Sum et al. (2022) [58] -cross-sectional-	Singapore A cohort of primary schoolchildren from Growing Up in Singapore Towards Healthy Outcomes	8 July 2020–5 September 2020 (after the end of strict universal movement restrictions) Self-administered electronic survey for parents and children.	N = 373 (F = 52.8%)	Range: 9–10.7 Mean age: 9.9 ± 0.4	Questionnaire	Collected from data reports of visits (BMI z scores were calculated using the WHO child growth standards)
Ten Velde et al. (2021) [59] -cross-sectional and longitudinal-	The Netherlands Representative sample of children in primary and secondary schools	Cohort A: February 2020 and May 2020. Cohort B: May–June 2019 and June 2020. Self-administered online questionnaires for parents or children > 12 yrs.	*Cohort A:* N = 102 (F = 57.6%) *Cohort B*: N = 131 (F = 56.5%)	Range: 4–18 *Cohort A:* 10.5 ± 3.6 *Cohort B:* 10.2 ± 0.9	Cohort A: Baecke questionnaire Cohort B: PA measured by accelerometer and Baecke questionnaire.	Cohort A: self-reported Cohort B: directly (Age- and sex-specific BMI z-scores were established (TNO Growth Calculator, TNO, The Hague, The Netherlands) WS was classified using the IOTF classification)
Thapar et al. (2024) [60] -longitudinal-	India Punjab 20 schools	Before: Sept 2018–February 2019 During pandemic: October 2020–March 2021	N = 308 (F = 53.6%)	12.9 ± 1.2	Subjectively with 3 questionnaires (GSHS, PAQ-C, Baecke) and objectively using an accelerometer for 7 days mounted on the right side of the waist in front of the right hip at all times except while sleeping or performing water activities such as swimming and bathing	Directly (BMI z-scores as per the 2007 WHO references)
Weihrauch-Blüher et al. (2023) [61] -cross-sectional-	Germany Selection of a representative sample of about 10,000 households in Germany	April/May 2022 Online interviews of parents by questionnaires	N = 1004 (F = 49.7%) 3–5 yrs: 24.4% 6–9 yrs: 23.3%, 10–12 yrs: 17.8% 13–14 yrs: 13.1% 15–17 yrs: 21.4%	Range: 3–17	Questionnaire	Pre-pandemic WS parent-reported
Yang et al. (2020) [62] -cross-sectional-	China Based on a national retrospective survey using a snowball sampling method.	Early May 2020 Questionnaires distributed via social media platforms on data before (23 December 2019, to 23 January 2020) and during L (24 January to 23 February 2020).	N = 2824 (F = 76%) high school students in a wider and older sample.	Mean age M + F: 17.5 ± 1.2 M: 17.5 ± 1.2 F: 17.5 ± 1.2	Questionnaire IPAQ-long form	Self-reported (OW and Ob defined based on the extended International Obesity Task Force recommended age sex- specific cut-offs corresponding to BMI ≥ 23 and ≥27 kg/m^2^ at age 18, respectively)
Yang et al. (2022) [63] -longitudinal-	China (Shanghai) Sampling from two junior high schools from each of the 16 districts	September–November 2019; September–November 2020 (one year after the pandemic outbreak) Self-completed questionnaires	N = 6047 (F = 48.9%)	Range: 11–16	Questionnaire	Directly (WS was based on age- and sex-specific BMI cut-offs according to the WHO. A Z-score of BMI > 1 was defined as OW and a Z-score > 2 was defined as Ob).
Yelizarova et al. (2022) [64] -cross-sectional-	Ukraine 79 localities in all regions of Ukraine	17 April–11 May 2020, and from 12 April–5 May 2021 Online questionnaires filled by parents.	N = 1091 (F = 46%) By school level: -primary school: N = 421 -secondary school: N = 521 -high school: N = 149. by year: -2020: N = 807 -2021: N = 284	Range: 6–18 Mean age Primary school M: 8.6 ± 1.4 F: 8.4 ± 1.2 Secondary school M: 13.0 ± 1.5 F: 12.9 ± 1.5 High school M: 16.3 ± 0.7 F: 16.3 ± 1.1 All schools M: 11.5 ± 3.0 F: 11.9 ± 3.1	Questionnaire GPAQ	Self-reported

Note: yrs: years; WS: weight status; UW: underweight; NW: normal weight; OW: overweight; Ob: obese; PA: physical activity; MVPA: moderate to vigorous physical activity; L: lockdown; M: males; F: females; VM: sum of the vector magnitude; GSHS: Global School-Based Student Health Survey; PAQ-C: Physical Activity Questionnaire for Older Children; GPAQ: Global Physical Activity Questionnaire; NR: not reported.

**Table 2:** Outcomes of the included studies.

Author (Year)	Changes in PA	Changes in BMI	Changes in WS	Main Outcomes
Abed Alah et al. (2024) [39]	PA decreased. Pre-COVID 2–4 days/week: 66.8% ≤1 day/week: ~15% During-COVID 2–4 days/week: 32% ≤1 day/week: 52% Screen time increased Pre-COVID: 17.4 ± 10.5 h/week (2.5 ± 1.5 h/day) During COVID 28.9 ± 14.1 h/week (4.1 ± 2.0 h/day)	BAZ increased. Pre-COVID: M + F: 0.61 ± 1.56 F: 0.6 ± 1.4 M: 0.7 ± 1.7 8–11 yrs: 0.4 ± 1.6 12–15 yrs: 0.9 ± 1.5 Post-COVID: M + F: 0.91 ± 1.57 F: 0.8 ± 1.5 M: 1.0 ± 1.7 8–11 yrs: 0.8 ± 1.6 12–15 yrs: 1.1 ± 1.5	Pre-COVID: M + F: OW 18%; Ob 22% F: OW 19%; Ob 19% M: OW 17%; Ob 24% 8–11 yrs: OW 15%; Ob 16% 12–15 yrs: OW 21%; Ob 28% Post-COVID: M + F: OW 22%; Ob 27% F: OW 22%; Ob 24% M: OW 22%; Ob 31% 8–11 yrs: OW 21%; Ob 25% 12–15 yrs: OW 24%; Ob 30%	The authors showed a significant reduction in PA, and significant increases in screen time and BAZ scores, resulting in a higher prevalence of obesity and overweight. In summary, nearly 50% of children/adolescents were OW/Ob, when schools reopened.
Al Hourani et al. (2022) [40]	Decreased: More than 50% had PA ≤ 1 h/day Screen time Children Before L: <1 h/d: 34% 1–2 h/d: 34% 2–3 h/d: 16% 3–4 h/d: 9.4% >4 h/d: 6.6% During L: <1 h/d: 16% 1–2 h/d: 20.1% 2–3 h/d: 21.3% 3–4 h/d: 17.6% >4 h/d: 25% Adolescents Before L: <1 h/d: 15.9% 1–2 h/d: 23.3% 2–3 h/d: 18.5% 3–4 h/d: 19.8% >4 h/d: 22.4% During L: <1 h/d: 5.6% 1–2 h/d: 12.1% 2–3 h/d: 10.8% 3–4 h/d: 22.4% >4 h/d: 49.1%	BAZ increased. Children: Before L: 0.32 ± 1.9 During L: 0.82 ± 1.9. Adolescents: Before L: 0.35 ± 1.43 During L: 0.54 ± 1.47	Children Before L: OW: 18% Ob: 16.7%, During L: OW: 24.1% (+6.1%) Ob: 24.1% (+7.4%) Adolescents Before L: OW: 23.3% Ob: 12.9% During L: OW: 20.7% (−2.6%) Ob: 16.4% (+3.5%) Severe thinness and thinness decreased or did not vary in children (−1.2 and −2.4, respectively) and adolescents (−0.9 and 0.0, respectively).	This study concluded that, due to school closures, there had been an alarming change in eating habits, weight gain, and physical inactivity among children and adolescents.
Al-Agha et al. (2022) [41]	Decreased. Never engaged in PA during weekdays: Pre-COVID: M: 17.6%; F: 23.7% During COVID: M: 37.5%; F: 40.5%.	BMI increased. Pre-COVID: -M: 19.85 ± 6.27 -F: 18.63 ± 6.10 During COVID: -M: 20.66 ± 6.73 -F: 19.51 ± 55.41	Increased. Pre-COVID: Ob: 6.20%; OW: 8.10% During COVID: Ob: 4.60%; OW: 12.00%	The COVID-19 pandemic has affected students’ PA and lifestyle by raising their OW and Ob risk.
Azrak et al. (2022) [42]	PA was reduced at 1st visit and increased at the 2nd one. Lockdown Group (LG) PA practice 1st visit vs. baseline Less: 59% The same: 14% More: 27% 2nd visit vs. 1st visit Less: 14% The same: 24% More: 62%	z-BMI were significantly higher in LG (1st visit) than in HCG In LG: z-BMI was significantly higher at 1st and 2nd visit vs. baseline.	In LG, the proportion of children with OW/Ob increased significantly from the baseline (43.5%) to 1st (56.5%) and 2nd visit (58.3%) and remained unchanged in HCG (41.0% to 42.5%). Ob children increased from 14.6% at baseline to 27.8% at 1st visit in LG (*p* = 0.009) and remained unchanged in HCG (14.2% to 17.2%).	The study found that weight gain continued even when there was an easing in the measures taken during the L, although sedentary behaviors decreased, suggesting that the pandemic L effects may be hard to reverse.
Basterfield et al. (2022) [43]	The mean time spent in sports clubs did not decrease significantly: -baseline: 231 min/wk -follow-up: 209 min/wk. The number of children taking part in a sports club decreased: -baseline: 87 -both time points: 61.	Mean BMI increased (+1.5 kg·m^−2^): -Baseline: 18.3 ± 3.3 -Follow-up: 19.8 ± 4.0. BMI z-score also increased significantly from baseline: -Baseline: 0.71 ± 1.19 -Follow-up: 0.95 ± 1.22. No sex difference was observed for BMI or BMI z-score.	110 children remained in the same weight category, 33 moved up a weight category, and 3 moved down. OW/Ob increased: from 33% to 47%.	Over the 12-month period that included the U.K.’s national L and extended school closures, there was an increased BMI and a decreased physical performance. Authors emphasized the importance of sports, PA, and fitness for recovery from COVID-19 L.
Benmerzoug et al. (2022) [44]	Sports practice decreased from 52.4% before L to 30.2% during L; screen time of more than 2 h/day raised from 3.3% before L to 5.5% during L. Sports change vs. BMI change: -decreased (n = 79), change in BMI = 1.08 ± 1.87 -no change (n = 178), change in BMI = 0.77 ± 1.56 -increased (n = 18), change in BMI = 0.44 ± 1.50. Regular activity change vs. BMI change: -decreased (n = 146), change in BMI = 0.98 ± 1.16 -no change (n = 99), change in BMI = 0.81 ± 1.73 -increased (n = 30), change in BMI = 0.98 ± 1.16. Screen time change vs. BMI change -decreased (n = 5), change in BMI = 0.32 ± 1.37 -no change (n = 88), change in BMI = 0.81 ± 1.62 -increased (n = 57), change in BMI = 0.77 ± 1.63.	BMI increased: -Pre-COVID: 18.77 ± 7.78 -During:19.61 ± 7.61 Change: 0.84 ± 1.65	Pre-COVID -UW: 7.6% -NW: 59.6% -OW: 16.7% -Ob: 16% During COVID -UW: 4.7% -NW: 50.2% -OW: 25.1% -Ob: 20%	Three months of restrictive measures during the COVID-19 L, home confinement, and school closures hurt the health of children: there was a decreased PA, an increased sedentary behavior, an increased screen time, and a gain in body weight and BMI.
Dallolio et al. (2022) [45]	Mean changes in MVPA (min) -weekly: −30.59 ± 120.87 (M: −52.09 ± 110.46; F: + 3.94 ± 85.89) -daily: −15.32 ± 16.21 (M: −19.54 ± 16.55; F: −8.32 ± 13.13) Sedentary activity (min/week) +1196.01 ± 381.49 (M: +1262.42 ± 386.60; F: +1086.08 ± 350.39) Step counts (n/week) −3152.53 ± 11,433.77 Light PA (min/week) = −16.16 ± 267.67 Moderate PA (min/week) −15.80 ± 65.86 Vigorous PA (min/week) −15.19 ± 46.06 PA levels (PAQ-C score) (N = 52) −0.87 ± 0.72 (M: −1.10 ± 0.80; F: −0.60 ± 0.52) Not meeting the recommended PA levels (<60min/day MVPA) before vs. during the pandemic: M: 43.75% vs. 85.42% F: 79.31% vs. 93.1%	BMI before vs. during pandemic Before: 17.49 ± 2.76 During: 17.91 ± 3.00	WS before vs. during pandemic -NW 71.1% vs. 68.4% -OW 21.1% vs. 26.3% -Ob 7.9% vs. 5.3%	The study highlights the reduction in PA during the COVID-19 epidemic, especially in boys, and the increase in sedentary habits.
Farello et al. (2022) [46]	*Perceived physical lifestyle* Children: pre- vs. post-L -More sedentary 2.7% vs. 45.4% -Sometimes sedentary 24.7% vs. 36.8% -Rarely sedentary 50.2% vs. 13.5% -Never sedentary 23.1% vs. 4.2%. Adolescents: pre- vs. post-L -More sedentary 5.5% vs. 68.4% -Sometimes sedentary 38.0% vs. 22.7% -Rarely sedentary 35.6% vs. 6.5% -Never sedentary 20.9% vs. 2.4% Training Children (pre- vs. during COVID): -No training 11.7% vs. 87.6% -1 time/week 9.8% vs. 4.0% -2 times/week 50.5% vs. 6.4% -3 times/week 24.5% vs. 1.3% -Daily 3.6% vs. 0.8%. Adolescents (pre- vs. during COVID): -No training 12.3% vs. 80.1% -1 time/week 4.1% vs. 6.9% -2 times/week 27.4% vs. 6.5% -3 times/week 47.3% vs. 2.7% -Daily 8.9% vs. 3.8%.	NR	*Perceived WS* Children (pre- vs. post-L): UW 6% vs. 3.5% NW 82.0% vs. 65.9% -OW 12.0% vs. 17.7% -Ob 0.0% vs. 0.6% Adolescents: (pre- vs. post L): -UW 5.2% vs. 5.3% -NW 82.7% vs. 81.7% -OW 11.6% vs. 12.4% -Ob 0.5% vs. 0.7%	Through the study, significant changes in the lifestyle of children and adolescents in Italy during the pandemic emerged with an increase in the incidence of Ob.
He et al. (2022) [47]	Daily PA decreased: 90.4 ± 50.9 min before vs. 83.1 ± 50.6 min during PA in 2019 vs. 2020: -M: 90.2 ± 50.8 vs. 83.9 ± 51.9 min -F: 90.6 ± 51.1 vs. 82.4 ± 49.4 min Screen time increased 85.3 ± 74.5 min before vs. 100.1 ± 73.9 min during Screen time in 2019 vs. 2020: -M: 87.8 ± 76.0 vs. 100.3 ± 73.6 min -F: 82.9 ± 73.0 vs. 99.9 ± 74.1 min	Mean BMI increased from 18.4 ± 3.2 kg/m^2^ before the pandemic to 18.5 ± 3.2 kg/m^2^ during the pandemic. BMI in 2019 vs. 2020: -M:18.4 ± 3.2 vs. 18.5 ± 3.3 kg/m^2^ -F: 18.4 ± 3.2 vs. 18.4 ± 3.2 kg/m^2^ -Primary school: 17.2 ± 2.3 vs. 17.2 ± 2.4 kg/m^2^ -Secondary school: 20.9 ± 3.5 vs. 21.0 ± 3.1 kg/m^2^	OW and Ob increased from 9.2% and 8.6% before the pandemic to 10.5% and 10.6% during the pandemic. Prevalence OW -2019 vs. 2020 -M: 10.1 vs. 12.4% -F: 8.2 vs. 8.6% -Primary school: 6.0 vs. 6.1% -Secondary school: 15.6 vs. 19.5% Prevalence Ob 2019 vs. 2020 -M: 8.3 vs. 12.1% -F: 8.9 vs. 9.2% -Primary school: 6.8 vs. 7.1% -Secondary school: 12.2 vs. 23.5%	The study suggests a trend of intensifying Ob in children during the COVID-19 pandemic, to which impaired weight-related behaviors may have largely concurred.
Kenđel Jovanović et al. (2021) [48]	PA reduction in MET (min/week) during L (M: Δ = 1468.9 ± 1107.8; F: Δ = 1234.1 ± 964.8) Comparisons before vs. during L: PA level M + F: -Low 19.3% vs. 75.6% -Moderate 79.3% vs. 23.6% -High 1.3 vs. 0.9%. M: -Low 16.3% vs. 75.7% -Moderate 81.7% vs. 23.3% -High 2.0% vs. 0.9%. F: -Low 22.0% vs. 75.4% -Moderate 77.3% vs. 23.8% -High 0.7% vs. 0.8%. MET-min/week M + F: 3813.7 ± 859.2 vs. 2470.1 ± 180.3 M: 3939.4 ± 894.9 vs. 2470.5 ± 386.6 F: 3703.9 ± 812.2 vs. 2469.8 ± 337.9. Organized Activities (Sports) M + F: -<2 d/week 30.2% vs. 30.7% -2–3 d/week 36.4% vs. 44.2% ->4 d/week 33.5% vs. 25.1%. M: -<2 d/week 24.7% vs. 25.8% -2–3 d/week 39.3% vs. 39.3% ->4 d/week 36.0% vs. 34.9%. F: -<2 d/week 34.9% vs. 35.0% -2–3 d/week 33.8% vs. 48.4% ->4 d/week 31.3% vs. 16.6%. Non-Organized Activities (Games) M + F: -<2 d/week 18.2% vs. 26.6% -2–3 d/week 34.3% vs. 28.0% ->4 d/week 47.5% vs. 45.5%. M: -<2 d/week 15.5% vs. 22.2% -2–3 d/week 32.6% vs. 28.3% ->4 d/week 52.0% vs. 49.5%. F: -<2 d/week 20.5% vs. 30.4% -2–3 d/week 35.8% vs. 27.6% ->4 d/week 43.6% vs. 42.0%. Two-thirds of participants said they used a PC/tablet/cell phone for more than 2 h/week, and over 90% looked at TV for <2 h/day.	BMI before vs. during M + F: 20.23 ± 3.87 vs. 20.89 ± 5.47 M: 20.53 ± 3.88 vs. 21.22 ± 6.95 F: 19.98 ± 3.84 vs. 20.60 ± 3.69.	WS before vs. during M + F: UW: 6.3% vs. 2.3% -NW: 72.9% vs. 73.7% -OW: 14.5% vs. 17.2% -Ob: 6.4% vs. 6.9%. M: -UW: 6.0% vs. 2.2% -NW: 71.4% vs. 72.1% -OW: 16.3% vs. 18.3% -Ob: 6.4% vs. 7.4%. F: -UW: 6.6% vs. 2.5% -NW: 74.2% vs. 75.0% -OW: 12.9% vs. 16.1% -Ob: 6.4% vs. 6.4%.	In Croatian schoolchildren, there was an increase in OW and Ob during the COVID-19 L. Their life habits changed: they became less physically active and their screen time increased.
Khamesan et al. (2024) [49]	Sedentary activities pre- vs. during L: 13% vs. 48% PA pre- vs. during L -Light intensity: 19% vs. 37% -Moderate intensity: 56% vs. 15% -Vigorous intensity: 12% vs. 0% Screen time pre- vs. during L -0–1 h: 22% vs. 3% -1–2 h: 41% vs. 8% -2–3 h:19% vs. 18% ->3 h: 18% vs. 71%	z-BMI score: -pre-L: −0.02 ± 1.64 -post-L: 0.36 ± 1.12 z-BMI change: Sex -F: 0.10 ± 0.85 -M: 0.90 ± 1.61 Age -10–12: 1.12 ± 1.62 -12–14: 0.19 ± 1.07 ->14: 0.49 ± 1.29	WS in pre- vs. post-L -Thinness/severe thin. (z-score < −2 SD) 8% vs. 1% -NW (−2SD < z-score < +1SD) 64% vs. 68% -OW/Ob (z-score > +1 SD) 28% vs. 31%.	Prolonged school closures due to the pandemic L worsened students’ health and lifestyle status. Children and adolescents significantly increased in BMI-for-age z-score during the quarantine period, especially among males and the 14–16 yrs age group. Those who decreased PA during school closures showed a significant rise in zBMI.
Łuszczki et al. (2021) [50]	Days in last week with PA ≥ 60 min: Pre: 3.89 ± 1.89 During: 3.30 ± 2.07 % of subjects met the recommended PA for children Pre: 12.3% During: 9.2%	Pre: 18.78 ± 3.83 During:18.46 ± 3.58	Pre (measured): UW 11.7% NW: 65.6% OW: 11.2% Ob: 11.5% During (self-reported): UW: 15.0% NW: 65.2% OW: 13.4% Ob: 6.4%	Children before the pandemic were more physically active than children during the pandemic.
Palermi et al. (2022) [51]	Did you do PA before lockdown? -yes 91.4% -no 8.6% Did you do PA during lockdown? -yes 70.6% -no 29.4%	BMI percentile -2019: 49.2 -2020: 65.2 -2021: 64.5 Mean BMI: -2019: 17.58 -2020: 18.48 -2021: 19.06	WS -UW 4.2% (in 2019), 0.6% (in 2020), 1.6% (in 2021). -NW 77.2% (in 2019), 63.2% (in 2020), 62.9% (in 2021). -OW 10.8% (in 2019), 17.6% (in 2020), 15.0% (in 2021). -Ob 7.8% (in 2019), 18.6% (in 2020), 20.5% (in 2021). Most parents (54.3%) said that their child’s weight increased during the quarantine.	The L resulting from the pandemic led to negative changes in childhood habits and lifestyle leading to weight gain as evidenced in a cohort of children in the Emilia Romagna region during a 3-year study period.
Park and Lim (2022) [52]	Changes (2019–2020) in Exercise M + F: 46.8% in 2019 vs. 43.7% in 2020 (difference: −3.1%) -12–15 yrs: 52.6% in 2019 vs. 48.0% in 2020 (difference: −4.6%) -16–18 yrs: 39.3% in 2019 vs. 38.4% in 2020 (difference: −0.9%) F: 29.0% in 2019 vs. 28.1% in 2020 (difference: −0.9%) -12–15 yrs: 35.5% in 2019 vs. 33.7% in 2020 (difference: −1.8%) -16–18 yrs: 15.0% in 2019 vs. 15.5% in 2020 (difference: 0.5%) M: 63.3% in 2019 vs. 58.1% in 2020 (difference: −5.2%) -12–15 yrs: 68.6% in 2019 vs. 61.3% in 2020 (difference: −7.3%) -16–18 yrs: 56.6% in 2019 vs. 54.2% in 2020 (difference: −2.3%) Changes (2019–2020) in moderate-intensity aerobic PA (for >60 min/day at least 3 times/wk) F: decreased slightly from 21.2% to 20.8% (non-Ob, 20.9–20.4%; Ob, 21.9–23.0%). M: decreased from 46.1% to 41.1% (non-Ob, 47.0–41.5%; Ob, 41.2–39.8%). Changes (2019–2020) in vigorous-intensity PA (for >20 min/day at least 3 times/wk) F: decreased in the 12–15 yrs group (21.9–19.6%); slight decrease in the 16–18 yrs group (12.8–12.6%). M: decreased in all age groups (12–15 yrs: 50.0–40.5%; 16–18 yrs: 38.2–34.5%) Changes in non-study sedentary hours M + F: 3.3 ± 2.2 in 2019 vs. 4.3 ± 2.8 in 2020 (difference: 1) -12–15 yrs: 3.4 ± 2.3 in 2019 vs. 4.4 ± 2.8 in 2020 (difference: 1) -16–18 yrs: 3.2 ± 2.0 in 2019 vs. 4.1 ± 2.7 in 2020 (difference: 1) F: 3.3 ± 2.2 in 2019 vs. 4.3 ± 2.8 in 2020 (difference: 1) -12–15 yrs: 3.4 ± 2.3 in 2019 vs. 4.4 ± 2.8 in 2020 (difference: 1) -16–18 yrs:3.2 ± 2.0 in 2019 vs. 4.1 ± 2.7 in 2020 (difference: 1) M: 3.3 ± 2.3 in 2019 vs. 4.2 ± 2.8 in 2020 (difference: 0.9) -12–15 yrs: 3.4 ± 2.3 in 2019 vs. 4.4 ± 2.8 in 2020 (difference: 1) -16–18 yrs: 3.2± 2.3 in 2019 vs. 4.1 ± 2.7 in 2020 (difference: 0.9)	BMI changes F: 2019: 20.7 ± 3.0 2020: 20.6 ± 3.1 M: 2019: 21.9 ± 3.9 2020: 22.3 ± 3.9 z-BMI changes F: 2019: −0.07 ± 1.14 2020: −0.11 ± 1.18 M: 2019: 0.12 ± 1.31 2020: 0.22 ± 1.35 BMI percentile changes F: 2019 <5: 7.1% ≥5 to <85: 76.1% ≥85 to <95: 8.8% ≥95: 8.1% 2020 <5: 8.5% ≥5 to <85: 74.5% ≥85 to <95: 8.6% ≥95: 8.4% M: 2019 <5: 7.1% ≥5 to <85: 68.6% ≥85 to <95: 10.5% ≥95: 13.8% 2020 <5: 7.1% ≥5 to <85: 65.7% ≥85 to <95: 11.6% ≥95: 15.6%	Changes in Ob prevalence M + F: -11.0% in 2019 vs. 12.1% in 2020 (difference:1.1%) -12–15 yrs: 9.5% in 2019 vs. 11.1% in 2020 (difference: 1.6%) -16–18 yrs: 13.0% in 2019 vs. 13.4% in 2020 (difference: 0.4%). F: 8.1% in 2019 vs. 8.4% in 2020 (difference: 0.3%) -12–15 yrs: 6.3% in 2019 vs. 6.6% in 2020 (difference: 0.3%) -16–18 yrs: 10.4% in 2019 vs. 10.6% in 2020 (difference: 0.1%). M: 13.8% in 2019 vs. 15.6% in 2020 (difference: 1.8%) -12–15 yrs: 12.5% in 2019 vs. 15.2% in 2020 (difference: 2.7%) -16–18 yrs: 15.4% in 2019 vs. 16.0% in 2020 (difference: 0.6%).	The study supports that the COVID-19 environment caused an increase in the prevalence of Ob in Korean adolescents aged 12–18 years. The decrease in PA due to the L and school closures especially affected middle school children.
Pujia et al. (2021) [53]-	PA during L: -More sedentary lifestyle: 79.5% -Less sedentary lifestyle: 10% -Did not know: 10.5% More sedentary lifestyle during L 5–9 yrs children with not increased weight vs. increased weight: 85% vs. 88% 10–14 yrs children with not increased weight vs. increased weight: 79% vs. 95%	BMI changes during L 5–9 yrs children with not increased weight vs. increased weight: −0.6 ± 0.8 vs. 0.5 ± 1 10–14 yrs children with not increased weight vs. increased weight: −1.2 ± 1 vs. 0.6 ± 0.9	NR	The study pointed out that body weight gain was associated with changes in stature and a higher consumption of dairy products and packaged sweet snacks in children, while in adolescents, it was associated with a higher intake of comfort foods and processed meats.
Ramos-Álvarez et al. (2021) [54]	PA pre-L vs. post-L -No PA: 4% vs. 32% -2–3 times/wk: 46% vs. 36% -4–5 times/wk: 40% vs. 26% -6–7 times/wk: 10% vs. 6%. Screen time for >60 min/day: -52% of the sample used the video console -50% the television -48% the computer -30% the tablet -26% the mobile phones	BMI changes M + F: -1st:19.56 ± 3.26 -2nd: 19.74 ± 3.44 -3rd: 17.95 ± 4.39 M: -1st:19.53 ± 3.00 -2nd: 19.77 ± 3.08 -3rd: 18.04 ± 4.11 F: -1st:19.61 ± 3.82 -2nd: 19.66 ± 4.17 -3rd: 17.77 ± 5.03	NR	The study highlights significant changes in the anthropometric parameters of 11–12-year-old boys and girls due to the impact of the L. Among the factors that generated this trend, the authors cited the decline in PA time during L.
Samigullin et al. (2024) [55]	Children were active for at least 60 min on 5 days/wk with a median PA per day of 96 min (mean 106 min). Changes during L PA -less or much less participation in sports (75%); -no changes in sports activities (16.3%); -more frequent participation (8.7%). Sitting behavior -more or much more time (55.8%); -no change (32.6%); -less or much less time (11.6%).	NR	Changes during L WS -no change (78.8%); -increased (15.2%); -decreased (3.9%).	Unlike other studies in the literature, the sample of children with high socioeconomic status in this study showed a decrease in weight during the pandemic, with a significant increase in UW.
So et al. (2022) [56]	MVPA (h/day) Pre: 0.47 ± 0.35 During: 0.42 ± 0.49 Schools partially reopened: 0.44 ± 0.33 Step count per day Pre: 10,969.33 ± 2492.15 During: 8472.51 ± 3295.39 Schools partially reopened: 9547.04 ± 2455.05	BMI (z-score) Pre: 0.17 ± 1.25 During: 0.24 ± 1.26 Schools partially reopened: 0.13 ± 1.29	Pre-pandemic: UW: 12 (2%) NW: 442 (77%) OW: 85 (15%) Ob: 34 (6%) During: UW: 2 (1%) NW: 111 (76%) OW: 21 (14%) Ob: 7 (5%) Schools partially reopened UW: 12 (4%) NW: 227 (77%) OW: 38 (13%) Ob: 15 (5%)	This study found that the COVID-19 pandemic is having potentially significant long-term impacts on the lifestyle and PA of school-aged children
Štveráková et al. (2021) [57]	PAQ-C score M + F: Pre: 2.69 ± 0.59 During: 2.30 ± 0.66 Mean difference (95% CI) 0.38 (0.24, 0.53) M: Pre: 2.69 (0.62) During: 2.32 (0.69) Mean difference (95% CI) 0.37 (0.14, 0.61) F: Pre: 2.68 (0.56) During: 29 (0.64) Mean difference (95% CI) 0.39 (0.20, 0.58) Mean number of steps = 7.767 steps/day (M: 9.255; F: 6.982).	M + F: 17.35 ± 2.85 M: 17.56 ± 3.06 F: 17.20 ± 2.70 Pre-COVID M + F: 17.92 (2.97) M: 18.46 ± 3.13 F: 17.36 ± 2.68	NR	L resulted in a significant reduction in PA in Czech children.
Sum et al. (2022) [58]	During L, children ceased outdoor play/exercise (34.27%)	Pre-L vs. Post-L BMI 17.25 ± 3.36 vs. 18.75 ± 3.89 (difference: 1.5) Pre-L vs. Post-L z-BMI 0.30 ± 1.50 vs. 0.57 ± 1.50 (difference: 0.27)	NR	All the adiposity measures were higher after the L than before L: the observed PA cessation was related to increased adiposity in schoolchildren one year after the L.
Ten Velde et al. (2021) [59]	Self-reported PA Cohort A (N = 102) 62% of children reported less total PA during the L than before. Total PA was 0.54 ± 0.92 points lower for boys and 0.22 ± 0.65 lower for girls Cohort B (N = 131) PA decreased in sports (−0.19 ± 0.64 points) but there was no difference in school and leisure time PA in comparison with the period before the pandemic. 54% of children reported a decrease in total PA. Total PA decreased by −0.34 ± 0.98 points in girls (no reduction in boys). Accelerometry before vs. during COVID Cohort B (N = 64) -Sedentary time (min/d): 420 ± 60 vs. 465 ± 70 -Light PA (min/d): 252 ± 34 vs. 218 ± 39 -MVPA (min/d): 65 ± 18 vs. 48 ± 18 -Counts per minute: 1236 ± 274 vs. 1003 ± 266 -Adhering to MVPA guideline of 60 min/d: 64% vs. 20%. Children adhering to guidelines in May 2019 spent 23 ± 20 min/d (30.5%) less in MVPA during COVID-19; other children spent 7 ± 9 min/d (14.9%) less in MVPA. Changes in PA during the pandemic in M vs. F: -Sedentary time (min/d): +52 ± 71 vs. +43 ± 66 -Light PA (min/d): −18 ± 38 vs. −44 ± 41 -MVPA (min/d): −15 ± 16 vs. −18 ± 20 -Counts per minute: −201 ± 203 vs. −252 ± 254.	z-BMI at baseline Cohort A (N = 49) −0.27 ± 1.14 Cohort B (N = 131) 0.24 ± 1.11	WS at baseline Cohort A (N = 49) -NW: 81.2% -OW: 18.9% Cohort B (N = 131) -NW: 80.8% -OW: 19.2%	The study’s findings indicated a decrease in the PA levels in school-age children during the pandemic and increased screen time—the children had become more sedentary. The authors also pointed out that the PA levels had still decreased and sedentariness increased even after schools and sports clubs reopened.
Thapar et al. (2024) [60]	60 min PA (d/wk) from PAQ-C: Pre: 4.8 ± 2.4 During: 3.9 ± 2.8 PA measured by accelerometer: (N = 37) MVPA ≥ 60 min Pre: 24 (65.5%) During: 21 (57.4%) Counts per minute: Pre: 798.2 ± 297.5 During: 776.2 ± 278.4	BMI z-score M + F (N = 308) Pre: −0.7 ±1.4 During: −0.5 ± 1.3 Mean difference: 95% CI: 0.2 (0.1–0.3) M (N = 143) Pre: −0.8 ± 1.5 During: −0.7 ± 1.5 F (N = 165) Pre: −0.6 ± 1.3 During: −0.4 ± 1.1	NR	The adolescents adopted healthier dietary practices but had decreased PA during the pandemic than before.
Weihrauch-Blüher et al. (2023) [61]	PA reduction: 44% PA increase: 7%. PA was reduced most in 10–12 yrs children (57%)	NR	Weight (W) changes at the pandemic beginning according to pre-pandemic WS: UW -slight W loss: 3% -no W change: 91% -slight excess W gain: 6% NW -slight W loss:6% -no W change: 81% -slight excess W gain: 12% -substantial excess W gain: 1% OW/Ob -substantial W loss: 6% -slight W loss: 13% no W change: 34% slight excess W gain: 35% substantial excess W gain: 12%	The study shows that adverse health effects related to the COVID-19 pandemic have emerged, especially in children 10–12 years of age and those from families with low incomes, resulting in worsening social inequality.
Yang et al. (2020) [62]	PA pre-COVID vs. L (median of hours/d) Active transport for commuting M + F:1.5 vs. 1.0 M: 1.5 vs. 1.0 F: 1.5 vs. 1.0 MVPA M + F: 1.5 vs. 1.5 M: 2.0 vs. 2.0 F: 1.5 vs. 1.5 Sedentary time (workdays) M + F: 3.5 vs. 4.0 M: 3.0 vs. 3.0 F: 3.5 vs. 4.2 Screen time M + F: 4.0 vs. 5.0 M: 4.0 vs. 4.0 F: 4.0 vs. 5.0. Changes in PA before and during L Active transport for commuting (Total) -increased 2.3% -constant 77.4% -decreased 20.3% MVPA -increased 3.5% -constant 80.4% -decreased 16.1% Sedentary time (workdays) -increased 36.4% -constant 38.6% -decreased 25.0% Screen time -increased 29.9% -constant 61.8% -decreased 8.3%	BMI pre-COVID vs. L M + F: 22.7 ± 6.7 vs. 23.8 ± 8.7 M: 21.7 ± 6.0 vs. 22.8 ± 7.5 F: 23.0 ± 6.9 vs. 24.1 ± 9.1	OW (BMI ≥ 23 kg/m^2^) pre-COVID vs. L Total: 26.6% vs. 30.3% M: 22.7% vs. 30.1% F: 27.8% vs. 30.4%. Ob (BMI ≥ 27 kg/m^2^) pre-COVID vs. L M + F: 16.0% vs. 18.8% M: 13.0% vs. 16.8% F: 17% vs. 19.5%.	The study showed changes in Ob and activity patterns among the participants in China: the prevalence of OW/Ob and Ob significantly increased in high school students. Patterns of all PA, sedentary, and screen use variables also changed with more youths increasing their sedentary and screen time
Yang et al. (2022) [63]	MVPA ≥ 60 min/day on all 7 days in 2019 vs. 2020 -M + F: 870 (14.4%) vs. 706 (11.7%) -M: 575 (18.6%) vs. 488 (15.8%) -F: 295 (10.0%) vs. 218 (7.4%) Days of MVPA ≥ 60 min/day in 2019 vs. 2020 -M + F: 3.5 ± 2.2 vs. 3.3 ± 2.1 -M: 3.8 ± 2.2 vs. 3.5 ± 2.2 -F: 3.2 ± 2.1 vs. 3.1 ± 2.0 Outdoor activities ≥2 h/day in 2019 vs. 2020 -M + F: 1990 (36.8%) vs. 1457 (26.9%) -M: 1065 (38.4%) vs. 794 (28.6%) -F: 925 (35.1%) vs. 663 (25.1%)	Changes in BMI (2019–2020) -M + F: 0.9 -M: 1.0 -F: 0.9 -11 yrs: 1.1 -12 yrs: 1.0 -13 yrs: 0.8 -14–16 yrs: 0.7	Ob prevalence in 2019 vs. 2020 -M + F: 14.2 vs. 15.4 -M: 20.9% vs. 23.1% -F: 7.1% vs. 7.2%	In China, the BMI and Ob prevalence of adolescents increased, mainly due to the increase in boys. PA and outdoor activities decreased, while screen time increased.
Yelizarova et al. (2022) [64]	PA duration during strict quarantine -total MVPA time 2020: 450.6 ± 339.7 min/wk 2021: 393.2 ± 342.8 min/wk -total LPA time 2020: 603.5 ± 381.2 min/wk 2021: 606.1 ± 355.0 min/wk Changes in PA Due to the 2021 L, there was a decrease in MVPA (-57.4 min per week) but not in LPA Children meeting 60 min/d of MVPA in 2020 vs. 2021 M: 47% vs. 33.4% F: 35.3% vs. 17.9%	BMI All schools M: -2020: 18.5 ± 3.7 -2021: 18.5 ± 3.5 F: -2020: 18.2 ± 3.2 -2021: 18.5 ± 3.2 Primary school M: -2020: 16.4 ± 2.9 -2021: 16.9 ± 2.9 F: -2020: 16.2 ± 2.7 -2021: 16.9 ± 3.3 Secondary school M: -2020: 19.8 ± 3.7 -2021: 19.5 ± 3.4 F: -2020: 18.9 ± 2.9 -2021: 19.2 ± 2.9 High school M: -2020: 20.8± 3.0 -2021: 21.2 ± 3.4 F: -2020: 20.2 ± 2.8 -2021:20.1 ± 2.4	WS -2020: OW/Ob = 18.6% -2021: OW/Ob = 22.4%	There was a significant decrease in the level of school-age children’s MVPA by 12.7% in 2021 in comparison with 2020. A 13.7 percent decrease in 2021 was also found in the percentage of children reaching the recommended levels of MVPA. Female sex, chronic diseases, OW/Ob, and non-participation in organized sports were some of the factors that negatively affected this trend.

Note: yrs: years; BAZ: BMI-for-age Z score; BMI-SDS: BMI standard deviation scores; z-BMI: BMI z-score; WS: weight status; UW: underweight; NW: normal weight; OW: overweight; Ob: obese; PA: physical activity; LPA: light physical activity; MVPA: moderate to vigorous physical activity; PAQ-C = Physical Activity Questionnaire for Older Children; Δ: difference; L: lockdown; M: males; F: females; NR: not reported.

**Table 3:** Quality assessment of the included studies by the Newcastle–Ottawa scale (NOS) with a relative range for each item. Higher scores indicate better quality research.

References in Alphabetical Order	Clarity of Stated Aim (0–2)	Sample Selection				Comparability		Outcome		NOS Score
		Sample Representativeness (0–2)	Sample Size (0–2)	Non-Respondents (0–2)	Ascertainment of the Exposure (0–2)	Control of Confounding Factors (0–1)	Comparability of Participants (0–1)	Assessment of the Outcome (0–2)	Statistical Tests (0–2)	(0–16)
Abed Alah et al. (2024) [39]	2	2	2	0	2	0	1	2	2	13 High
Al Hourani et al. (2022) [40]	2	1	2	0	2	0	1	1	1	10 Moderate
Al-Agha et al. (2022) [41]	1	2	0	0	1	0	1	2	1	8 Low
Azrak et al. (2022) [42]	2	0	1	1	1	1	1	2	2	11 Moderate
Basterfield et al. (2022) [43]	2	0	1	0	2	1	1	2	2	11 Moderate
Benmerzoug et al. (2022) [44]	1	1	0	0	1	0	1	1	2	7 Low
Dallolio et al. (2022) [45]	2	2	0	0	2	0	0	2	1	9 Moderate
Farello et al. (2022) [46]	2	2	1	0	2	0	1	1	1	10 Moderate
He et al. (2022) [47]	2	2	1	2	0	0	1	2	2	12 Moderate
Kenđel Jovanović et al. (2021) [48]	2	1	2	0	1	0	1	2	2	11 Moderate
Khamesan et al. (2024) [49]	1	2	0	0	1	0	1	2	1	8 Low
Łuszczki et al. (2021) [50]	2	2	2	0	1	0	1	2	1	11 Moderate
Palermi et al. (2022) [51]	2	1	1	0	1	0	1	2	1	9 Moderate
Park and Lim (2022) [52]	2	2	2	1	1	0	1	1	2	12 Moderate
Pujia et al. (2021) [53]	2	1	1	0	2	1	1	1	2	11 Moderate
Ramos-Álvarez et al. (2021) [54]	2	0	0	0	0	0	0	2	2	6 Low
Samigullin et al. (2024) [55]	2	1	0	0	2	0	1	2	2	10 Moderate
So et al. (2022) [56]	2	2	1	0	2	1	0	1	1	10 Moderate
Štveráková et al. (2021) [57]	2	0	1	0	2	0	1	1	1	8 Low
Sum et al. (2022) [58]	2	1	1	0	2	1	1	2	2	12 Moderate
Ten Velde et al. (2021) [59]	1	1	0	0	2	0	1	2	2	9 Moderate
Thapar et al. (2024) [60]	2	2	2	2	2	0	1	2	1	14 High
Weihrauch-Blüher et al. (2023) [61]	2	2	1	0	1	0	1	1	1	9 Moderate
Yang et al. (2020) [62]	1	2	1	0	2	0	0	1	1	8 Low
Yang et al. (2022) [63]	2	2	2	2	1	0	1	2	2	14 High
Yelizarova et al. (2022) [64]	2	1	2	0	2	1	1	1	2	12 Moderate

## Data Availability

Not applicable.

## Supplementary Materials

The following supporting information can be downloaded at: https://www.mdpi.com/article/10.3390/children12020178/s1, Table S1: PRISMA 2020 for abstract checklist; Table S2: PRISMA 2020 checklist. References [87] is cited in the supplementary materials
